# Lymphocyte-to-Monocyte Ratio and Clinical Outcomes in Cholangiocarcinoma: A Systematic Review and Meta-Analysis

**DOI:** 10.3390/diagnostics12112655

**Published:** 2022-11-01

**Authors:** Giuseppe Dotto-Vasquez, Andrea K. Villacorta-Ampuero, Juan R. Ulloque-Badaracco, Enrique A. Hernandez-Bustamante, Esteban A. Alarcón-Braga, Percy Herrera-Añazco, Vicente A. Benites-Zapata, Adrian V. Hernandez

**Affiliations:** 1 Escuela de Medicina, Universidad Peruana de Ciencias Aplicadas, Lima 15023, Peru; 2 Sociedad Científica de Estudiantes de Medicina de la Universidad Nacional de Trujillo, Trujillo 13011, Peru; 3 Grupo Peruano de Investigación Epidemiológica, Unidad para la Generación y Síntesis de Evidencias en Salud, Universidad San Ignacio de Loyola, Lima 15012, Peru; 4 Sociedad Científica de Estudiantes de Medicina de la Universidad Peruana de Ciencias Aplicadas, Lima 15023, Peru; 5 Escuela de Enfermería, Universidad Privada San Juan Bautista, Lima 15067, Peru; 6 Instituto de Evaluación de Tecnologías en Salud e Investigación—IETSI, EsSalud, Lima 14072, Peru; 7 Unidad de Investigación para la Generación y Síntesis de Evidencias en Salud, Vicerrectorado de Investigación, Universidad San Ignacio de Loyola, Lima 14072, Peru; 8 Unidad de Revisiones Sistemáticas y Meta-análisis, Guías de Práctica Clínica y Evaluaciones de Tecnología Sanitaria, Vicerrectorado de Investigación, Universidad San Ignacio de Loyola, Lima 15012, Peru; 9 Health Outcomes, Policy, and Evidence Synthesis Group, University of Connecticut School of Pharmacy, Mansfield, CT 06269, USA

**Keywords:** cholangiocarcinoma, lymphocyte–monocyte ratio, survival, meta-analysis

## Abstract

Lymphocyte-to-Monocyte ratio (LMR) has shown an association with survival outcomes in several oncological diseases. This study aimed to evaluate the association between LMR and clinical outcomes for cholangiocarcinoma patients. A systematic review and meta-analysis were performed to assess the association between LMR values and overall survival (OS), disease-free survival (DFS), recurrence-free survival (RFS) and time to recurrence (TTR) in cholangiocarcinoma patients. We used Hazard ratio (HR) and their 95% confidence interval (CI) as a measure of effect for the random effect model meta-analysis. The Newcastle–Ottawa Scale was used for quality assessment. The Egger test and funnel plot were developed for approaching publication bias. A total of 19 studies were included in this study (n = 3860). The meta-analysis showed that cholangiocarcinoma patients with low values of LMR were associated with worse OS (HR: 0.82; 95% CI: 0.71–0.96; I^2^ = 86%) and worse TTR (HR: 0.71; 95% CI: 0.58–0.86; I^2^ = 0%). DFS and RFS also were evaluated; however, they did not show statistically significant associations. Low LMR values were associated with a worse OS and TTR.

## 1. Introduction

Cancer refers to cells that grow out of control and invade other tissues [1]. Cholangiocarcinoma (CCA), or bile duct cancer, is a malignant and lethal adenocarcinoma of the hepatobiliary system that can be divided into three anatomical regions: intrahepatic, perihilar (extrahepatic) and distal. Each anatomical subtype has a clinical presentation and therapeutic approach [2]. The most frequent cancer found in the bile duct bifurcation is called perihilar cholangiocarcinoma or Klatskin tumor. However, intrahepatic cholangiocarcinoma (ICC) is the second most common liver malignancy, characterized by its late diagnosis and fatal outcome, ranking behind hepatocellular carcinoma (HCC) [3]. Cholangiocarcinoma represents 3% of all gastrointestinal tumors and 10–15% of all hepatobiliary tumors [4]. This cancer is common in Asian countries such as Thailand [5] and South Korea but rare in countries like Brazil and Costa Rica [6]. However, despite its low prevalence and incidence, recent studies have shown that ICC’s incidence and mortality rates are increasing [7].

The etiology remains uncertain, but it is known that there is an association with chronic inflammation of the bile ducts, such as primary sclerosing cholangitis, chronic hepatitis and cirrhosis [8]. Most patients are asymptomatic in the early stages of the disease until advanced stages; therefore, their diagnosis is late. Most people receive a cholangiocarcinoma diagnosis after cancer has already spread to other organs. The life expectancy is usually poor, and it will depend on the location of cancer and its stage. Bile duct cancer survival is 50% at one year, 20% at two years and 10% at three years [1].

Because of the suggested role of inflammation in the genesis and prognosis of cancer, several inflammatory response markers have been studied, such as the neutrophil-to-lymphocyte ratio (NLR), which is associated with the prognosis of different types of cancers [9,10]. Lymphocyte-to-monocyte ratio (LMR) is another inflammatory marker that has shown prognostic value in different types of cancers and may have a prognostic value in patients with cholangiocarcinoma [11,12,13]. Although studies have been published that have evaluated the role of LMR in clinical outcomes of patients with cholangiocarcinoma, the available evidence has not been systematized to the best of our knowledge. Therefore, the aim of this research is to evaluate the association between LMR and clinical outcomes in patients with cholangiocarcinoma.

## 2. Methods

### 2.1. Research Question and Study Design

This systematic review was conducted to answer the research question based on the Population, Exposure, Comparison and Outcome (PECO) strategy: Do patients with cholangiocarcinoma (P) and low values of LMR (E) have worse clinical outcomes (O) than patients with cholangiocarcinoma and high values of LMR (C)?

### 2.2. Register and Report Guideline

This study was registered on the International Prospective Register of Systematic Reviews (PROSPERO) with code CRD42021290302, and the Preferred Reporting Items for Systematic Reviews and Meta-analysis (PRISMA) statement was used for reporting [14].

### 2.3. Search Strategy and Data Sources

The search strategy for this systematic review was built following the Peer Review of Electronic Search Strategies (PRESS) checklist with no language or date restriction [15]. At first, it was built for Pubmed with MeSH and free terms and afterwards, it was adapted to the other databases. On 30 November, 2021, an advanced search was performed for retrieving studies assessing the association between LMR and clinical outcomes in patients with cholangiocarcinoma through the following peer review databases: PubMed, Scopus, Web of Science and The Cochrane Library (See Search Strategy in Supplementary S1). In addition, a hand-search was carried out in preprint databases (Medrixv and ResearchSquare).

### 2.4. Eligibility Criteria, Study Selection and Data Extraction

Inclusion criteria were studies: (i) with case-control or cohort design, (ii) conducted in adult patients (≥18 years old) with a confirmed diagnosis of cholangiocarcinoma and (iii) that assessed the association between LMR and clinical outcomes in cholangiocarcinoma patients. Studies without all eligibility criteria and duplicates were excluded. The primary outcome was Overall Survival (OS) and Disease-Free Survival (DFS). Recurrence Free Survival (RFS) and Time to Recurrence (TTR) were secondary outcomes (see definitions of outcomes for each study in Supplementary Table S1). Rayyan QCRI software was used for study selection and removing duplicates [16]. First, four authors (GD-V, AKV-A, JRU-B and EAH-B) screened the retrieved records independently by titles and abstracts. Then, these authors assessed the remaining records independently by full text. Any conflicts in the screening process were resolved by consensus of all authors. Finally, four authors’ sheets (GD-V, AKV-A, JRU-B and EAH-B) collected data from included studies in a preset data extraction Microsoft Excel ©. Collected data were: first author, study title, publication date, study design, study location, population baseline characteristics (number of participants, age, sex, comorbidities, stratified sample data), Hazard Ratio (HR) and corresponding 95% confidence interval (CI) as association measure between LMR values and OS, RFS, DFS or TTR.

### 2.5. Quality Assessment

Quality assessment was evaluated independently with the Newcastle–Ottawa Scale (NOS) by two authors (GD-V and AKV-A) and scores were categorized as: low risk of bias (≥7 stars), moderate risk of bias (4–6 stars) and high risk of bias (≤3 stars) [17].

### 2.6. Data Synthesis and Publication Bias

Statistical analysis was performed using Review Manager 5.4 (RevMan 5.4). Estimates for HR and their 95% CI were pooled by generic inverse variance, and due to anticipated heterogeneity, a random-effects meta-analysis was performed. Heterogeneity analysis was assessed using the I^2^ test and Cochran’s Q-statistic. Test values were categorized as: severe heterogeneity (≥60%) and mild heterogeneity (<60%). A *p*-value of <0.1 was considered statistically significant. Additionally, a subgroup analysis was developed by study location treatment (after curative resection vs. without curative resection) and according to cut-off values (LMR ≥ 3.5 vs. LMR < 3.5), and the interaction test *p*-value per subgroup analysis was reported. Finally, sensitivity analyses were performed using the low risk of bias studies only. Publication bias was assessed through funnel plots and Egger’s test, and a *p*-value < 0.1 was considered indicative of publication bias.

## 3. Results

### 3.1. Study Selection

**Figure 1:**
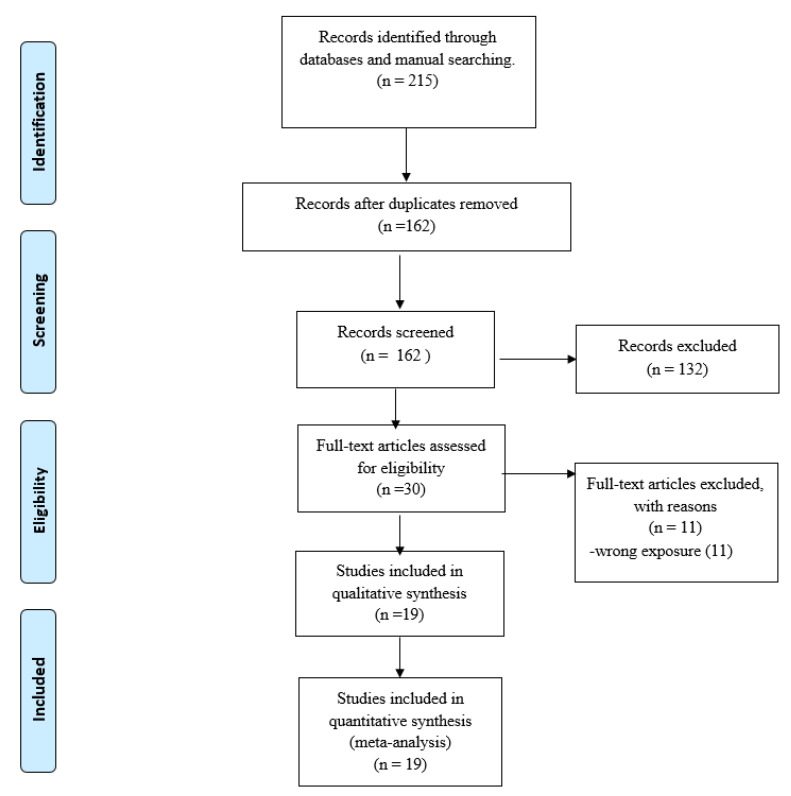
PRISMA Flow Diagram.

We identified 215 articles, leaving 162 studies after eliminating duplicates. Next, the screening by titles and abstracts excluded 132 studies because of lack of relevance and left 30 studies for the full-text review. Then, 11 full-text articles were excluded because of wrong exposure. Finally, a total of 19 articles were included in the meta-analysis [18,19,20,21,22,23,24,25,26,27,28,29,30,31,32,33,34,35,36]. A flow diagram of the literature search is shown in Figure 1.

### 3.2. Study Characteristics

We included 19 articles, giving us a total of 21 cohort studies, because two articles analyzed data from two different cohorts. All studies evaluated OS, four evaluated RFS and three evaluated DFS and TTR. These were studies carried out in four countries, 15 studies in China, four in Japan, one in South Korea and one in Italy. There was a total of 3860 participants, of which 2333 were men. The age ranges of the participants were between 20 and 87 years old. However, three studies did not provide us with the participants’ ages. In addition, the range of medians was provided by 18 studies with a range of 42 to 70. According to the TNM stage, it was found that 1441 patients were in stages I and II, while 744 were in stages III and IV. Finally, most studies focused on patients with intrahepatic cholangiocarcinoma (17 studies). On the other hand, 16 studies evaluated optimal LMR cut-off values for OS, RFS, DFS and TTR, ranging from 2.1 to 8. The NOS identified that eight cohorts had a moderate risk of bias, and only 13 had a low risk of bias (see Supplementary Table S2). The characteristics of each study are summarized in Table 1.

### 3.3. Association between LMR and OS in Cholangiocarcinoma Patients

**Figure 2:**
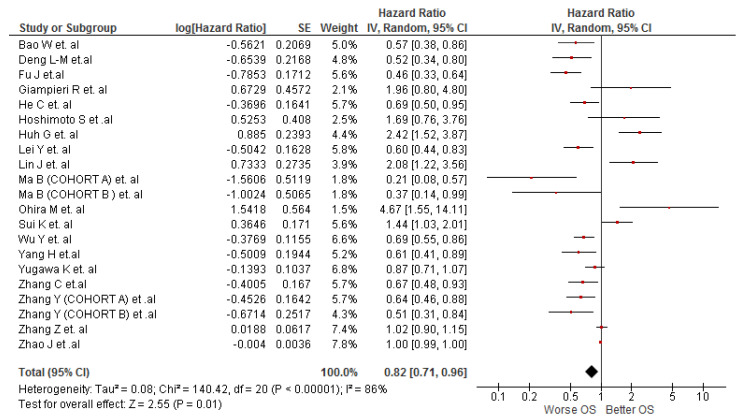
Association of LMR and OS in patients with cholangiocarcinoma.

This association was evaluated by 21 cohort studies (n = 3860) and meta-analysis showed that cholangiocarcinoma patients with low values of LMR were associated with a worse OS (HR: 0.82; 95% CI: 0.71–0.96; I^2^ = 85%) (Figure 2). Due to high heterogeneity, subgroup analyses were carried out according to cut-off values, study location and treatment. In the subgroups analysis by cut-off values, we found that LMR values lower than 3.5 showed a statistically significant association with a worse OS (HR: 0.58; 95% CI: 0.46–0.74; I^2^ = 57%). On the other hand, LMR values greater than or equal to 3.5 did not show a statistically significant association with OS (HR: 1.07; 95% CI: 0.73–1.55; I^2^ = 87%) (see Supplementary Figure S1). The curative resection subgroup retained the association with OS (HR: 0.72; 95% CI: 0.57–0.93; I^2^ = 85%) and patients without curative resection lost the statistically significant association (HR: 1.02; 95% CI: 0.81–1.30; I^2^ = 80%) (see Supplementary Figure S2). Regarding subgroup analysis by study location, just the Chinese studies subgroup (HR: 0.68; 95% CI: 0.58–0.81; I^2^ = 87%) retained the statistically significant association with OS (Supplementary Figure S3). The sensitivity analysis showed a significant decrease of heterogeneity in the association of low values of LMR and worse OS (HR: 0.64; 95% CI: 0.55–0.74; I^2^ = 41%) (see Supplementary Figure S4).

### 3.4. Association between LMR and DFS in Cholangiocarcinoma Patients

**Figure 3:**
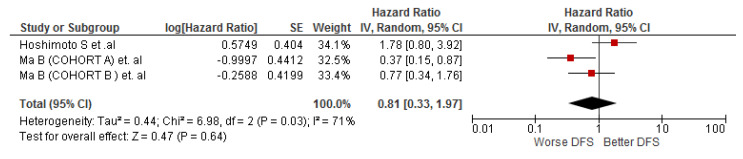
Association of LMR and DFS in patients with cholangiocarcinoma.

The association between LMR and DFS was evaluated by three cohort studies (n = 227), and the meta-analysis did not show statistically significant results for this association in cholangiocarcinoma patients (HR: 0.81; 95% CI: 0.33–1.97; I^2^ = 71%) (Figure 3).

### 3.5. Association between LMR and RFS in Cholangiocarcinoma Patients

**Figure 4:**
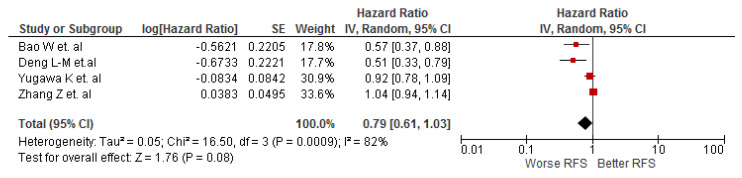
Association of LMR and RFS in patients with cholangiocarcinoma.

The association between LMR and RFS was evaluated by four cohort studies (n = 551), and the meta-analysis did not show statistically significant results for this association in cholangiocarcinoma patients (HR: 0.79; 95% CI: 0.61–1.03; I^2^ = 82%) (Figure 4).

### 3.6. Association between LMR and TTR in Cholangiocarcinoma Patients

**Figure 5:**
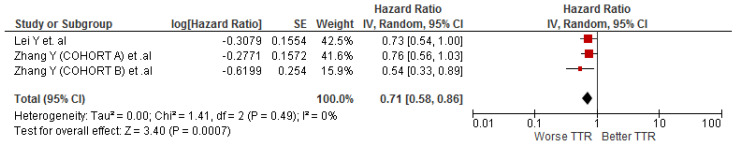
Association of LMR and TTR in patients with cholangiocarcinoma.

The association between LMR and TTR was evaluated by three cohort studies (n = 748) and the meta-analysis showed that cholangiocarcinoma patients with low values of LMR were associated with worse TTR (HR: 0.71; 95% CI: 0.58–0.86; I^2^ = 0%) (Figure 5).

## 4. Discussion

The main results of our study show that patients with cholangiocarcinoma who have low LMR values were associated with worse OS and TTR. Inflammation is one of the main contributors to the malignant transformation of cells by creating reactive oxygen species and activating cell signaling pathways that promote cell proliferation and limit the degree of apoptosis [37,38]. It also influences cancer progression through its effect on the cellular components of the immune system. Additionally, although the overall effects of cellular immunity on cancer progression are still debated, a chronic state of immune stimulation is associated with a poor prognosis [39]. In that sense, different markers associated with inflammation have been studied as prognostic inflammatory markers of different types of cancers, such as neutrophil-to-lymphocyte ratio (NLR) or platelet-to-lymphocyte ratio (PLR), which have shown usefulness in urogenital and gastrointestinal cancers [9,10,40].

The LMR is composed of two important factors in tumor progression. The first is the immune response to the tumor shown by the number of lymphocytes, potentially including tumor-infiltrating lymphocytes [41]. These induce a DNA damage response, leading to apoptosis or excessive autophagy [42]. In contrast, monocytes associated with malignant tissue, commonly called tumor-associated macrophages, are drivers of cancer progression due to their contribution to angiogenesis and lymphangiogenesis [43,44]. This mechanism results in increased tumor cell proliferation capacity, increased intravascular fluid flow and increased rates of distant metastasis [45]. In this regard, several systematic reviews have shown that a high LMR value was associated with longer disease-free days and recurrence-free survival in patients with hepatocellular carcinoma and pancreatic cancer [11]. Likewise, a high value was associated with a better prognosis in head and neck cancer [46]. Similarly, a low value was associated with worse OS in patients with esophageal cancer [47], lower OS and progression-free survival in patients with lung cancer [13] and worse prognosis in patients with renal [48] and breast cancer [49].

In patients with cholangiocarcinoma, inflammation has been shown to play an essential role in both genesis and progression. Regardless of its etiology, most risk factors for cholangiocarcinoma cause inflammation or cholestasis [50]. Chronic inflammation leads to increased exposure of cholangiocytes to inflammatory mediators, causing progressive mutations in tumor suppressor genes, proto-oncogenes and DNA mismatch repair genes [50]. The accumulation of bile acids from cholestasis leads to a reduced pH, increased apoptosis, and activation of mediators that stimulate cell proliferation, migration and survival [50]. Additionally, the presence and maintenance of an inflammatory microenvironment at the primary tumor site plays a vital role in the development and metastasis through mechanisms that activate tumor vasculature and improve angiogenesis and lymphangiogenesis [51].

Although our results are promising, significance was not found in all the outcomes evaluated, as occurred in other types of cancers. For example, in patients with hepatocarcinoma [11], LMR was not associated with OS, and in patients with renal carcinoma [48], a low LMR values were not associated with OS and DFS. Although our study does not assess the reasons, it is likely to be related to some of the patient’s characteristics that influence the outcomes of other types of cancers. Similarly, in patients with pancreatic and breast cancer, the prognostic value of LMR was observed in subgroups such as ethnicity, surgery treatment, stage of the disease and LMR cut-off value < 3 [12], or Asian populations, triple negative patients and patients with non-metastatic disease and mixed stage, respectively [49].

In contrast, the prognostic value appeared to be influenced by histologic type in lung cancer [13] or some histopathologic features in renal carcinoma [48]. These findings suggest that some patient characteristics may influence the association depending on the clinical outcome assessed.

Our results show enough evidence to recommend a low LMR value as a marker associated with worse TTR and OS in patients with cholangiocarcinoma. Our study is the first systematic review and meta-analysis that evaluates these associations. Furthermore, we performed sensitivity analyses considering the biases, which increases the robustness our results. Our findings allow us to suggest a potential marker of low-cost in cholangiocarcinoma that will allow health workers to prioritize or individualize management strategies in patients with low LMR values. However, since some characteristics of patients or cancer may affect the association with some clinical outcomes, it is suggested to design studies that consider different subgroups of patients [13,48].

### Limitations

This study has several limitations, which should be considered for future research. First, most of the studies found in this systemic review were developed on the Asian continent, preventing us from identifying good comparisons between different ethnic groups. Secondly, the studies did not adjust LMR values with confounding variables that influenced the results of the study. Sociodemographic and clinical factors must be adjusted to improve accuracy in different populations. In the third place, due to lack of information in the included studies, the values of specificity, sensitivity and an optimal cut-off point could not be estimated in a meta-analysis to predict different outcomes in patients with cholangiocarcinoma. In fourth place, we found a high heterogeneity between the included studies, which is attributed to the high risk of bias of several studies. Finally, most of the studies included are retrospective, so information bias is more likely due to the use of information from usual clinical practice, so it is necessary to carry out more prospective studies, where researchers can have a better control of measurements in patients.

## 5. Conclusions

Low LMR values are associated with a worse OS and TTR. In addition, no statistically significant associations were found between LMR values and DFS or RFS. It is necessary to carry out prospective studies to corroborate the findings of this research.

## Figures and Tables

**Table 1:** Characteristics of the included studies.

Author	Year	Study Location	Median Follow-Up Time	Participants (Male)	Median/Mean Age (IQR/SD)	Type of Cholangiocarcinoma Evaluated	Outcome	HR(95% CI), *p*-Value	Cut-Off	TNM Stage (I–II/III–IV)
*Wu Y et al. [18]*	2019	China	29.1 months	123 (67)	57 (11)	Intrahepatic	Overall Survival	0.686 (0.547–0.819), *p* < 0.05	3.42	38/85
*He C et al. [36]*	2021	China	NR	292 (181)	56 (20–77)	Intrahepatic	Overall Survival	0.691 (0.501–0.953), *p* < 0.05	4.06	107/185
*Lin J et al. [20]*	2019	China	NR	123 (65)	60 (31–85)	Intrahepatic	Overall Survival	2.082 (1.218–3.558), *p* < 0.05	3.62	99/24
*Huh G et al. [22]*	2020	South Korea	35.4 months	137 (83)	64 (57–72)	Intrahepatic	Overall Survival	2.423 (1.516–3.875), *p* < 0.05	3.5	NR/NR
*Ohira M et al. [23]*	2021	Japan	NR	52 (41)	61 (39–82)	Intrahepatic	Overall Survival	4.673 (1.547–20.165), *p* < 0.05	4.36	35/17
*Yang H et.al [26]*	2019	China	44 months	299 (181)	NR	Intrahepatic	Overall Survival	0.606 (0.414–0.885), *p* < 0.05	4.45	226/73
*Fu J et.al [33]*	2021	China	NR	446 (295)	54.36 (10.71)	Intrahepatic	Overall Survival	0.465 (0.326–0.663), *p* < 0.005	2.48	NR/NR
*Sui K et al. [24]*	2020	Japan	27.6 months	273 (164)	70 (9.4)	Intrahepatic	Overall Survival	1.44 (1.03–2.43), *p* < 0.05	3.7	NR/NR
*Giampieri R et al. [30]*	2021	Italy	NR	45 (NR)	NR	Mixed	Overall Survival	1.96 (0.80–4.8), *p* = 0.138	2.1	NR/NR
*Zhao J et al. [32]*	2021	China	NR	468 (282)	58 (51–65)	Intrahepatic	Overall Survival	0.996 (0.989–1.003), *p* = 0.302	NR	NR/NR
*Zhang C et al. [35]*	2016	China	NR	187 (117)	58 (12)	Intrahepatic	Overall Survival	0.67 (0.483–0.931), *p* < 0.05	3	NR/NR
*Bao W et al. [19]*	2021	China	28.7 months	178 (85)	64 (10)	Intrahepatic	Overall Survival	0.57 (0.38–0.87), *p* < 0.05	3	126/52
							Recurrence-free survival	0.57 (0.37–0.86), *p* < 0.05		
*Zhang Z et al. [21]*	2020	China	NR	128 (70)	56 (10)	Intrahepatic	Overall Survival	1.019 (0.903–1.151), *p* = 0.757	NR	53/75
							Recurrence-free survival	1.039 (0.943–1.146), *p* = 0.435		
*Yugawa K et al. [27]*	2021	Japan	NR	78 (55)	66 (39–87)	Intrahepatic	Overall Survival	0.87 (0.71–1.71), *p* = 0.1354	NR	NR/NR
							Recurrence-free survival	0.92 (0.78–1.06), *p* = 0.2414		
*Ma B (COHORT A) et al. [25]*	2021	Tianjin, China	NR	72 (41)	59 (32–76)	Intrahepatic	Overall Survival	0.21 (0.077–0.569), *p* < 0.05	2.65	NR/NR
							Disease Free Survival	0.368 (0.155–0.874), *p* < 0.05		
*Ma B (COHORT B) et al.* [25]	2021	Weifang, China	25.1 months	102 (57)	49 (28–77)	Intrahepatic	Overall Survival	0.367 (0.136–0.993), *p* < 0.05	2.7	NR/NR
							Disease Free Survival	0.772 (0.339–1.758), *p* = 0.537		
*Hoshimoto S et al. [34]*	2019	Japan	NR	53 (31)	70 (50–87)	Distal	Overall Survival	1.691 (0.760–3.764), *p* = 0.198	4.633	50/3
							Disease Free Survival	1.777 (0.805–3.925), *p* = 0.155	3.208	
*Deng L-M et al.* [29]	2021	China	29.3 months	167 (83)	63 (9)	Intrahepatic	Overall Survival	0.52 (0.34–0.8), *p* < 0.05	3.13	116/51
							Recurrence-free survival	0.51 (0.33–0.78), *p* < 0.05		
*Lei Y et al. [28]*	2020	China	44 months	322 (194)	NR	Intrahepatic	Overall Survival	0.604 (0.439–0.831), *p* < 0.05	4.45	248/74
							Time to recurrence	0.735 (0.542–0.997), *p* < 0.05		
*Zhang Y (COHORT A) et al. [31]*	2019	China	44 months	322 (194)	58 (27–81)	Intrahepatic	Overall Survival	0.636 (0.461– 0.878), *p* < 0.05	4.45	248/74
							Time to recurrence	0.758 (0.557–1.032), *p* = 0.079		
*Zhang Y (COHORT B) et al. [31]*	2019	China	38.3 months	104 (47)	42 (33–56)	Intrahepatic	Overall Survival	0.511 (0.312–0.837), *p* < 0.05	4.45	95/31
							Time to recurrence	0.538 (0.327–0.884), *p* < 0.05		

NR: Not Reported.

## Data Availability

Not applicable.

## Supplementary Materials

The following supporting information can be downloaded at: https://www.mdpi.com/article/10.3390/diagnostics12112655/s1, Supplementary S1: Search Strategy. Table S1: Outcome definitions for each study; Table S2: Newcastle–Ottawa quality assessment scale for included studies; Figure S1: Subgroup analysis according to the cut-off values (≥3.5 vs. <3.5) of the association between LMR and OS in patients with cholangiocarcinoma; Figure S2: Subgroup analysis according to the treatment of the association between LMR and OS in patients with cholangiocarcinoma; Figure S3: Subgroup analysis according to the origin country of the association between LMR and OS in patients with cholangiocarcinoma; Figure S4: Sensitivity analysis according to risk of bias of the association between LMR and OS in patients with cholangiocarcinoma; Figure S5: Funnel Plot of the studies that evaluated the association between LMR values and OS.
