# Supplementary material for: Lymphocyte-to-Monocyte Ratio and Clinical Outcomes in Cholangiocarcinoma: A Systematic Review and Meta-Analysis
Source: Diagnostics (Basel). 2022 Nov 1;12(11):2655. doi: 10.3390/diagnostics12112655 (PMC9689307; doi:10.3390/diagnostics12112655)

Lymphocyte-to-monocyte ratio and clinical outcomes in Cholangiocarcinoma: A systematic review and meta-analysis

SUPPLEMENTARY MATERIAL

S1 : Search Strategy

PUBMED

Lymphocyte (#1)

Lymphocytes [MH] OR Lymphocyte [MH] OR Lymphoid Cells [MH] OR Cell, Lymphoid [MH] OR Cells, Lymphoid [MH] OR Lymphoid Cell [MH] OR Lymphocytes [TIAB] OR Lymphocyte [TIAB] OR “Lymphoid Cells” [TIAB] OR “Lymphoid Cell” [TIAB] OR Lymphocytes [OT] OR Lymphocyte [OT] OR “Lymphoid Cells” [OT] OR “Lymphoid Cell” [OT]

Monocyte (#2)

Monocyte [MH] OR Monocytes [MH] OR Monocyte [OT] OR Monocytes [OT] OR Monocyte [TIAB] OR Monocytes [TIAB]

Lymphocyte to monocyte ratio (#3)

“Lymphocyte/Monocite ratio” [OT] OR “Lymphocyte/Monocite index” [OT] OR “Ratio Lymphocyte/Monocite” [OT] OR “Index Lymphocyte/Monocite” [OT] OR “Lymphocyte to Monocite ratio” [OT] OR “Lymphocyte-to Monocite ratio” [OT] OR “Lymphocyte to-Monocite ratio” [OT] OR “Lymphocyte-to-Monocite ratio” [OT] OR “Lymphocyte to Monocite index” [OT] OR “Lymphocyte-to Monocite index” [OT] OR “Lymphocyte to-Monocite index” [OT] OR “Lymphocyte-to-Monocite index” [OT] OR “Ratio Lymphocyte to Monocite” [OT] OR “Ratio Lymphocyte-to Monocite” [OT] OR “Ratio Lymphocyte to-Monocite” [OT] OR “Ratio Lymphocyte-to-Monocite” [OT] OR

“Index Lymphocyte to Monocite” [OT] OR “Index Lymphocyte-to Monocite” [OT] OR “Index Lymphocyte to-Monocite” [OT] OR “Index Lymphocyte-to-Monocite” [OT] OR “Lymphocyte-monocyte ratio” [OT] OR “Lymphocyte-monocyte index” [OT] OR “Ratio Lymphocyte-monocyte” [OT] OR “Index Lymphocyte-monocyte” [OT] OR LMR [OT]

Cholangiocarcinoma (#4)

Cholangiocarcinoma [MH] OR Cholangiocarcinomas [MH] OR Cholangiocellular Carcinoma [MH] OR Carcinoma, Cholangiocellular [MH] OR Carcinomas, Cholangiocellular [MH] OR Cholangiocellular Carcinomas [MH] OR Extrahepatic Cholangiocarcinoma [MH] OR Cholangiocarcinoma, Extrahepatic [MH] OR Cholangiocarcinomas, Extrahepatic [MH] OR Extrahepatic Cholangiocarcinomas [MH] OR Intrahepatic Cholangiocarcinoma [MH] OR Cholangiocarcinoma, Intrahepatic [MH] OR Cholangiocarcinomas, Intrahepatic [MH] OR Intrahepatic Cholangiocarcinomas [MH] OR Cholangiocarcinoma [TIAB] OR Cholangiocarcinomas [TIAB] OR Cholangiocellular Carcinoma [TIAB] OR “Cholangiocellular Carcinomas” [TIAB] OR “Extrahepatic Cholangiocarcinoma” [TIAB] OR “Extrahepatic Cholangiocarcinomas” [TIAB] OR “Intrahepatic Cholangiocarcinoma” [TIAB] OR “Intrahepatic Cholangiocarcinomas” [TIAB] OR Cholangiocarcinoma [OT] OR Cholangiocarcinomas [OT] OR Cholangiocellular Carcinoma [OT] OR “Cholangiocellular Carcinomas” [OT] OR “Extrahepatic Cholangiocarcinoma” [OT] OR “Extrahepatic Cholangiocarcinomas” [OT] OR “Intrahepatic Cholangiocarcinoma” [OT] OR “Intrahepatic Cholangiocarcinomas” [OT]

Search Formula :

((#1 AND #2) OR #3) AND #4

SCOPUS

(((TITLE-ABS-KEY ("Lymphocytes" OR "Lymphocyte" OR "Lymphoid Cells" OR "Cell, Lymphoid" OR "Cells, Lymphoid" OR "Lymphoid Cell" OR "Lymphocytes" OR "Lymphocyte" OR "Lymphoid Cells" OR "Lymphoid Cell" OR "Lymphocytes" OR "Lymphocyte" OR "Lymphoid Cells" OR "Lymphoid Cell")) AND (TITLE-ABS-KEY ("Monocyte" OR "Monocytes" OR "Monocyte" OR "Monocytes" OR "Monocyte" OR "Monocytes"))) OR (ALL ("Lymphocyte/Monocite ratio" OR "Lymphocyte/Monocite index" OR "Ratio Lymphocyte/Monocite" OR "Index Lymphocyte/Monocite" OR "Lymphocyte to Monocite ratio" OR "Lymphocyte-to Monocite ratio" OR "Lymphocyte to-Monocite ratio" OR "Lymphocyte-to-Monocite ratio" OR "Lymphocyte to Monocite index" OR "Lymphocyte-to Monocite index" OR "Lymphocyte to-Monocite index" OR "Lymphocyte-to-Monocite index" OR "Ratio Lymphocyte to Monocite" OR "Ratio Lymphocyte-to Monocite" OR "Ratio Lymphocyte to-Monocite" OR "Ratio Lymphocyte-to-Monocite" OR "Index Lymphocyte to Monocite" OR "Index Lymphocyte-to Monocite" OR "Index Lymphocyte to-Monocite" OR "Index Lymphocyte-to-Monocite" OR "Lymphocyte-monocyte ratio" OR "Lymphocyte-monocyte index" OR "Ratio Lymphocyte-monocyte" OR "Index Lymphocyte-monocyte" OR "LMR"))) AND (TITLE-ABS-KEY ("Cholangiocarcinoma" OR "Cholangiocarcinomas" OR "Cholangiocellular Carcinoma" OR "Carcinoma, Cholangiocellular" OR "Carcinomas, Cholangiocellular" OR "Cholangiocellular Carcinomas" OR "Extrahepatic Cholangiocarcinoma" OR "Cholangiocarcinoma, Extrahepatic" OR "Cholangiocarcinomas, Extrahepatic" OR "Extrahepatic Cholangiocarcinomas" OR "Intrahepatic Cholangiocarcinoma" OR "Cholangiocarcinoma, Intrahepatic" OR "Cholangiocarcinomas, Intrahepatic" OR

"Intrahepatic Cholangiocarcinomas" OR "Cholangiocarcinoma" OR "Cholangiocarcinomas" OR "Cholangiocellular Carcinoma" OR "Cholangiocellular Carcinomas" OR "Extrahepatic Cholangiocarcinoma" OR "Extrahepatic Cholangiocarcinomas" OR "Intrahepatic Cholangiocarcinoma" OR "Intrahepatic Cholangiocarcinomas" OR "Cholangiocarcinoma" OR "Cholangiocarcinomas" OR "Cholangiocellular Carcinoma" OR "Cholangiocellular Carcinomas" OR "Extrahepatic Cholangiocarcinoma" OR "Extrahepatic Cholangiocarcinomas" OR "Intrahepatic Cholangiocarcinoma" OR "Intrahepatic Cholangiocarcinomas"))

WEB OF SCIENCE

(((ALL=("Lymphocytes" OR "Lymphocyte" OR "Lymphoid Cells" OR "Cell, Lymphoid" OR "Cells, Lymphoid" OR "Lymphoid Cell" OR "Lymphocytes" OR "Lymphocyte" OR "Lymphoid Cells" OR "Lymphoid Cell" OR "Lymphocytes" OR "Lymphocyte" OR "Lymphoid Cells" OR "Lymphoid Cell")) AND ALL=("Monocyte" OR "Monocytes" OR "Monocyte" OR "Monocytes" OR "Monocyte" OR "Monocytes")) OR ALL=("Lymphocyte/Monocite ratio" OR "Lymphocyte/Monocite index" OR "Ratio Lymphocyte/Monocite" OR "Index Lymphocyte/Monocite" OR "Lymphocyte to Monocite ratio" OR "Lymphocyte-to Monocite ratio" OR "Lymphocyte to-Monocite ratio" OR "Lymphocyte-to-Monocite ratio" OR "Lymphocyte to Monocite index" OR "Lymphocyte-to Monocite index" OR "Lymphocyte to-Monocite index" OR "Lymphocyte-to-Monocite index" OR "Ratio Lymphocyte to Monocite" OR "Ratio Lymphocyte-to Monocite" OR "Ratio Lymphocyte to-Monocite" OR "Ratio Lymphocyte-to-Monocite" OR "Index Lymphocyte to Monocite" OR "Index Lymphocyte-to Monocite" OR "Index Lymphocyte to-Monocite" OR "Index Lymphocyte-to-Monocite" OR "Lymphocyte-monocyte ratio" OR "Lymphocyte-monocyte index" OR "Ratio Lymphocyte-monocyte" OR "Index Lymphocyte-

monocyte" OR "LMR")) AND ALL=("Cholangiocarcinoma" OR "Cholangiocarcinomas" OR "Cholangiocellular Carcinoma" OR "Carcinoma, Cholangiocellular" OR "Carcinomas, Cholangiocellular" OR "Cholangiocellular Carcinomas" OR "Extrahepatic Cholangiocarcinoma" OR "Cholangiocarcinoma, Extrahepatic" OR "Cholangiocarcinomas, Extrahepatic" OR "Extrahepatic Cholangiocarcinomas" OR "Intrahepatic Cholangiocarcinoma" OR "Cholangiocarcinoma, Intrahepatic" OR "Cholangiocarcinomas, Intrahepatic" OR "Intrahepatic Cholangiocarcinomas" OR "Cholangiocarcinoma" OR "Cholangiocarcinomas" OR "Cholangiocellular Carcinoma" OR "Cholangiocellular Carcinomas" OR "Extrahepatic Cholangiocarcinoma" OR "Extrahepatic Cholangiocarcinomas" OR "Intrahepatic Cholangiocarcinoma" OR "Intrahepatic Cholangiocarcinomas" OR "Cholangiocarcinoma" OR "Cholangiocarcinomas" OR "Cholangiocellular Carcinoma" OR "Cholangiocellular Carcinomas" OR "Extrahepatic Cholangiocarcinoma" OR "Extrahepatic Cholangiocarcinomas" OR "Intrahepatic Cholangiocarcinoma" OR "Intrahepatic Cholangiocarcinomas")

COCHRANE LIBRARY

("Lymphocytes" OR "Lymphocyte" OR "Lymphoid Cells" OR "Cell, Lymphoid" OR "Cells, Lymphoid" OR "Lymphoid Cell" OR "Lymphocytes" OR "Lymphocyte" OR "Lymphoid Cells" OR "Lymphoid Cell" OR "Lymphocytes" OR "Lymphocyte" OR "Lymphoid Cells" OR "Lymphoid Cell"):ti,ab,kw AND ("Monocyte" OR "Monocytes" OR "Monocyte" OR "Monocytes" OR "Monocyte" OR "Monocytes"):ti,ab,kw OR ("Lymphocyte to Monocite ratio" OR "Lymphocyte-to Monocite ratio" OR "Lymphocyte to-Monocite ratio" OR "Lymphocyte-to-Monocite ratio" OR "Lymphocyte to Monocite index" OR "Lymphocyte-to Monocite index" OR "Lymphocyte to-Monocite index" OR "Lymphocyte-to-Monocite index" OR "Ratio Lymphocyte to Monocite" OR "Ratio

Lymphocyte-to Monocite" OR "Ratio Lymphocyte to-Monocite" OR "Ratio Lymphocyte-to-Monocite" OR "Index Lymphocyte to Monocite" OR "Index Lymphocyte-to Monocite" OR "Index Lymphocyte to-Monocite" OR "Index Lymphocyte-to-Monocite" OR "Lymphocyte-monocyte ratio" OR "Lymphocyte-monocyte index" OR "Ratio Lymphocyte-monocyte" OR "Index Lymphocyte-monocyte" OR "LMR") AND ("Cholangiocarcinoma" OR "Cholangiocarcinomas" OR "Cholangiocellular Carcinoma" OR "Carcinoma, Cholangiocellular" OR "Carcinomas, Cholangiocellular" OR "Cholangiocellular Carcinomas" OR "Extrahepatic Cholangiocarcinoma" OR "Cholangiocarcinoma, Extrahepatic" OR "Cholangiocarcinomas, Extrahepatic" OR "Extrahepatic Cholangiocarcinomas" OR "Intrahepatic Cholangiocarcinoma" OR "Cholangiocarcinoma, Intrahepatic" OR "Cholangiocarcinomas, Intrahepatic" OR "Intrahepatic Cholangiocarcinomas" OR "Cholangiocarcinoma" OR "Cholangiocarcinomas" OR "Cholangiocellular Carcinoma" OR "Cholangiocellular Carcinomas" OR "Extrahepatic Cholangiocarcinoma" OR "Extrahepatic Cholangiocarcinomas" OR "Intrahepatic Cholangiocarcinoma" OR "Intrahepatic Cholangiocarcinomas" OR "Cholangiocarcinoma" OR "Cholangiocarcinomas" OR "Cholangiocellular Carcinoma" OR "Cholangiocellular Carcinomas" OR "Extrahepatic Cholangiocarcinoma" OR "Extrahepatic Cholangiocarcinomas" OR "Intrahepatic Cholangiocarcinoma" OR "Intrahepatic Cholangiocarcinomas"):ti,ab,kw

OVID MEDLINE

(“Lymphocytes” OR “Lymphocyte” OR “Lymphoid Cells” OR “Cell, Lymphoid” OR “Cells, Lymphoid” OR “Lymphoid Cell” OR “Lymphocytes” OR “Lymphocyte” OR “Lymphoid Cells” OR “Lymphoid Cell” OR “Lymphocytes” OR “Lymphocyte” OR “Lymphoid Cells” OR “Lymphoid Cell”).af. AND (“Monocyte” OR “Monocytes” OR “Monocyte” OR “Monocytes” OR “Monocyte” OR “Monocytes”).af. OR (“Lymphocyte to Monocite ratio” OR “Lymphocyte-to Monocite ratio” OR “Lymphocyte to-Monocite ratio” OR “Lymphocyte-to-Monocite ratio” OR “Lymphocyte to Monocite index” OR “Lymphocyte-to Monocite index” OR “Lymphocyte to-Monocite index” OR “Lymphocyte-to-Monocite index” OR “Ratio Lymphocyte to Monocite” OR “Ratio Lymphocyte-to Monocite” OR “Ratio Lymphocyte to-Monocite” OR “Ratio Lymphocyte-to-Monocite” OR “Index Lymphocyte to Monocite” OR “Index Lymphocyte-to Monocite” OR “Index Lymphocyte to-Monocite” OR “Index Lymphocyte-to-Monocite” OR “Lymphocyte-monocyte ratio” OR “Lymphocyte-monocyte index” OR “Ratio Lymphocyte-monocyte” OR “Index Lymphocyte-monocyte” OR “LMR”).af. AND (“Cholangiocarcinoma” OR “Cholangiocarcinomas” OR “Cholangiocellular Carcinoma” OR “Carcinoma, Cholangiocellular” OR “Carcinomas, Cholangiocellular” OR “Cholangiocellular Carcinomas” OR “Extrahepatic Cholangiocarcinoma” OR “Cholangiocarcinoma, Extrahepatic” OR “Cholangiocarcinomas, Extrahepatic” OR “Extrahepatic Cholangiocarcinomas” OR “Intrahepatic Cholangiocarcinoma” OR “Cholangiocarcinoma, Intrahepatic” OR “Cholangiocarcinomas, Intrahepatic” OR “Intrahepatic Cholangiocarcinomas” OR

“Cholangiocarcinoma” OR “Cholangiocarcinomas” OR “Cholangiocellular Carcinoma” OR “Cholangiocellular Carcinomas” OR “Extrahepatic Cholangiocarcinoma” OR “Extrahepatic Cholangiocarcinomas” OR “Intrahepatic Cholangiocarcinoma” OR “Intrahepatic Cholangiocarcinomas” OR “Cholangiocarcinoma” OR “Cholangiocarcinomas” OR “Cholangiocellular Carcinoma” OR “Cholangiocellular Carcinomas” OR “Extrahepatic Cholangiocarcinoma” OR “Extrahepatic Cholangiocarcinomas” OR “Intrahepatic Cholangiocarcinoma” OR “Intrahepatic Cholangiocarcinomas”).af.

Table S1 : Outcome definitions for each study

Author	Outcome	Definition
**Wu Y et al. [18]**	OS	The time between radical surgery and mortality.
**He C et al. [36]**	OS	The date of surgery to the date of death and tumor progression, respectively, or the last follow-up.
**Lin J et al. [20]**	OS	The interval between the date of resection and the date of death or the last follow-up.
**Huh G et al. [22]**	OS	The time from chemotherapy initiation until death or the last follow-up.
**Ohira M et al. [23]**	OS	NR
**Yang H et.al [26]**	OS	The interval between the dates of partial hepatectomy and death or between the dates of partial hepatectomy and the last observation.
**Fu J et.al [33]**	OS	The interval between the date of LR to the date censored, the date of the patient's death, or last follow-up.
**Sui K et al. [24]**	OS	The surgery date until death is related to ICC.
**Giampieri R et al.[30]**	OS	NR
**Zhao J et al. [32]**	OS	NR
**Zhang C et al. [35]**	OS	The interval between the date of a definitive diagnosis and death or between the date of a definitive diagnosis and the last observation of surviving patients
**Bao W et al. [19]**	OS	The date of surgery to the date of patient's death or the last follow-up date
	RFS	The date of surgery to the date of the first recurrence or last follow-up
**Zhang Z et al. [21]**	OS	The first day after hepatectomy to the ICC-related death.
	RFS	The first day after hepatectomy to the recurrence of ICC- or ICC-related death
**Yugawa K et al. [27]**	OS	The time from the date of surgery to the date of the last follow-up or death.
	RFS	NR
**Ma B et. Al [25]**	OS	The date of first diagnosis of ICC to the date of death or last follow-up

	DFS	The period between the date of surgery and the date of first recurrence or last follow-up
**Hoshimoto S et al. [34]**	OS	The time period from the date of surgery to either the date of death or the date of the last follow-up, whichever occurred first
	DFS	The time period from the surgery date to the date of recurrence, last follow-up, or death, whichever occurred first.
**Deng L-M et al. [29]**	OS	The date of surgery to the date of patient death or last follow-up
	RFS	The surgery date to the date of first ICC recurrence, death, or last follow-up visit.
**Lei Y et al. [28]**	OS	NR
	TTR	NR
**Zhang Y et. al [31]**	OS	The dates of operation to the dates of death or the dates of the last follow-up
	TTR	The interval between the dates of surgery and the first recurrence or from the dates of operation to the dates of last follow-up (for the patients without recurrence).

NR : NOT REPORTED

Table S2. Newcastle - Ottawa quality assessment scale for included studies

NEWCASTLE - OTTAWA QUALITY ASSESSMENT SCALE FOR COHORT STUDIES										
STUDY	**SELECTION**				**COMPARABILITY**	**OUTCOME**				
	**Representativeness of the exposed cohort**	**Selection of the non-exposed cohort**	**Ascertainment of exposure**	**Demonstration that outcome of interest was not present at start of study**	**Comparability of Cohorts on the Basis of the Design or Analysis Maximum : ☆☆**	**Assessment of outcome**	**Was follow-up long enough for outcomes to occur**	**Adequacy of follow up of cohorts**	**SCORE**	**Evidence quality**
Wu Y et al. [18]	☆	☆	☆	☆	☆	☆	☆	☆	*8*	Low Risk of bias
He C et al. [36]		☆	☆	☆	☆	☆	☆	☆	*7*	Low Risk of bias
Lin J et al. [20]	☆		☆	☆	☆	☆		☆	*6*	Moderate risk of bias
Huh G et al. [22]		☆	☆	☆	☆	☆	☆		*6*	Moderate risk of bias
Ohira M et al. [23]	☆		☆	☆	☆			☆	*5*	Moderate risk of bias
Yang H et.al [26]	☆	☆	☆	☆	☆☆	☆	☆	☆	*9*	Low Risk of bias
Sui K et al. [24]	☆			☆		☆	☆		*4*	Moderate risk of bias
Giampieri R et al.[30]	☆	☆	☆	☆	☆	☆	☆	☆	*8*	Low Risk of bias
Zhao J et al. [32]	☆	☆	☆	☆			☆		*5*	Moderate risk of bias
Zhang C et al. [35]	☆	☆	☆		☆	☆	☆	☆	*7*	Low Risk of bias
Bao W et al. [19]	☆	☆	☆	☆	☆		☆	☆	*7*	Low Risk of bias

Zhang Z et al. [21]	☆	☆	☆			☆	☆	☆	*6*	Moderate risk of bias
Yugawa K et al. [27]	☆	☆		☆		☆	☆		*5*	Moderate risk of bias
Ma B (COHORT A) et. Al [25]	☆	☆	☆	☆	☆	☆		☆	*7*	Low Risk of bias
Ma B (COHORT B) et. Al [25]	☆	☆	☆	☆	☆	☆		☆	*7*	Low Risk of bias
Fu J et.al [33]	☆		☆	☆	☆			☆	*5*	Moderate risk of bias
Hoshimoto S et al. [34]	☆	☆	☆	☆	☆☆	☆	☆	☆	*9*	Low Risk of bias
Deng L-M et al. [29]	☆	☆	☆	☆	☆	☆	☆	☆	*8*	Low Risk of bias
Lei Y et al. [28]	☆	☆	☆		☆	☆	☆	☆	*7*	Low Risk of bias
Zhang Y (COHORT A) et .al [31]	☆	☆	☆		☆☆	☆	☆	☆	*8*	Low Risk of bias
Zhang Y (COHORT B) et .al [31]	☆	☆	☆		☆☆	☆	☆	☆	*8*	Low Risk of bias

Figure S1. Subgroup analysis according to the cut-off values (≥3.5 vs <3.5) of the association between LMR and OS in patients with cholangiocarcinoma

Study or Subgroup	log[Hazard Ratio]	SE	Weight	Hazard Ratio IV, Random, 95% CI
**1.2.1 ≥3.5**				
He C et. al	-0.3696	0.1641	6.6%	0.69 [0.50, 0.95]
Hoshimoto S et .al	0.5253	0.408	4.2%	1.69 [0.76, 3.76]
Huh G et. al	0.885	0.2393	5.9%	2.42 [1.52, 3.87]
Lei Y et. al	-0.5042	0.1628	6.6%	0.60 [0.44, 0.83]
Lin J et. al	0.7333	0.2735	5.5%	2.08 [1.22, 3.56]
Ohira M et. al	1.5418	0.564	3.0%	4.67 [1.55, 14.11]
Sui K et. al	0.3646	0.171	6.6%	1.44 [1.03, 2.01]
Yang H et.al	-0.5009	0.1944	6.3%	0.61 [0.41, 0.89]
Zhang Y (COHORT A) et .al	-0.4526	0.1642	6.6%	0.64 [0.46, 0.88]
Zhang Y (COHORT B) et .al	-0.6714	0.2517	5.7%	0.51 [0.31, 0.84]
**Subtotal (95% CI)**			**57.1%**	**1.07 [0.73, 1.55]**
Heterogeneity: Tau² = 0.29; Chi² = 67.21, df = 9 (P < 0.00001); I² = 87%				
Test for overall effect: Z = 0.34 (P = 0.74)				
**1.2.2 <3.5**				
Bao W et. al	-0.5621	0.2069	6.2%	0.57 [0.38, 0.86]
Deng L-M et.al	-0.6539	0.2168	6.1%	0.52 [0.34, 0.80]
Fu J et.al	-0.7657	0.1812	6.5%	0.47 [0.33, 0.66]
Giampieri R et. al	0.6729	0.4572	3.7%	1.96 [0.80, 4.80]
Ma B (COHORT A) et. al	-1.5606	0.5119	3.3%	0.21 [0.08, 0.57]
Ma B (COHORT B) et. al	-1.0024	0.5065	3.4%	0.37 [0.14, 0.99]
Wu Y et. al	-0.3769	0.1155	7.0%	0.69 [0.55, 0.86]
Zhang C et.al	-0.4005	0.167	6.6%	0.67 [0.48, 0.93]
**Subtotal (95% CI)**			**42.9%**	**0.58 [0.46, 0.74]**
Heterogeneity: Tau² = 0.06; Chi² = 16.09, df = 7 (P = 0.02); I² = 57%				
Test for overall effect: Z = 4.37 (P < 0.0001)				
**Total (95% CI)**			**100.0%**	**0.81 [0.63, 1.03]**
Heterogeneity: Tau² = 0.21; Chi² = 98.71, df = 17 (P < 0.00001); I² = 83%				
Test for overall effect: Z = 1.69 (P = 0.09)				
Test for subgroup differences: Chi² = 7.10, df = 1 (P = 0.008), I² = 85.9%				

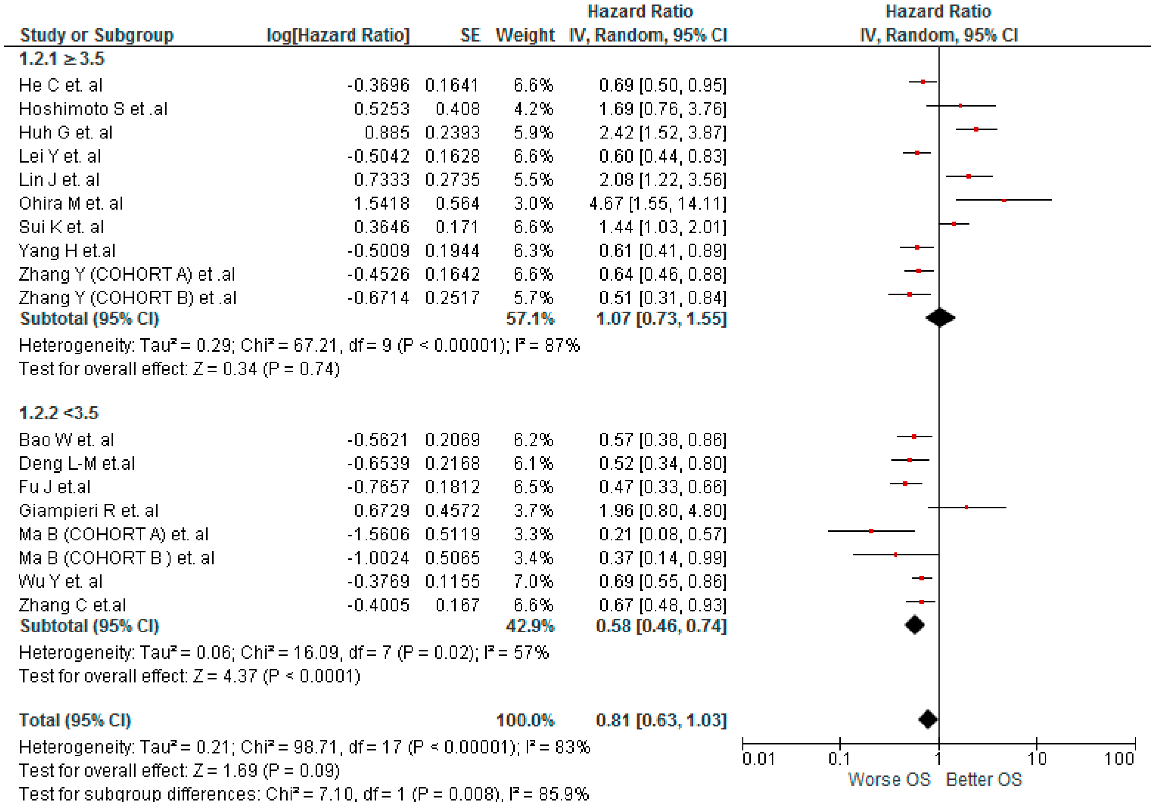

Figure S2. Subgroup analysis according to the treatment of the association between LMR and OS in patients with cholangiocarcinoma

Study or Subgroup	log[Hazard Ratio]	SE	Weight	Hazard Ratio IV, Random, 95% CI
**1.3.1 After Curative Resection**				
Bao W et. al	-0.5621	0.2069	5.0%	0.57 [0.38, 0.86]
Deng L-M et.al	-0.6539	0.2168	4.8%	0.52 [0.34, 0.80]
Fu J et.al	-0.7853	0.1712	5.6%	0.46 [0.33, 0.64]
Lei Y et. al	-0.5042	0.1628	5.8%	0.60 [0.44, 0.83]
Lin J et. al	0.7333	0.2735	3.9%	2.08 [1.22, 3.56]
Ma B (COHORT A) et. al	-1.5606	0.5119	1.8%	0.21 [0.08, 0.57]
Ma B (COHORT B) et. al	-1.0024	0.5065	1.8%	0.37 [0.14, 0.99]
Ohira M et. al	1.5418	0.564	1.5%	4.67 [1.55, 14.11]
Sui K et. al	0.3646	0.171	5.6%	1.44 [1.03, 2.01]
Wu Y et. al	-0.3769	0.1155	6.6%	0.69 [0.55, 0.86]
Yang H et.al	-0.5009	0.1944	5.2%	0.61 [0.41, 0.89]
Zhang Y (COHORT A) et .al	-0.4526	0.1642	5.7%	0.64 [0.46, 0.88]
Zhang Y (COHORT B) et .al	-0.6714	0.2517	4.3%	0.51 [0.31, 0.84]
Zhang Z et. al	0.0188	0.0617	7.4%	1.02 [0.90, 1.15]
**Subtotal (95% CI)**			**65.0%**	**0.72 [0.56, 0.93]**
Heterogeneity: Tau² = 0.16; Chi² = 87.47, df = 13 (P < 0.00001); I² = 85%				
Test for overall effect: Z = 2.56 (P = 0.01)				
**1.3.2 Without Curative Surgery**				
Giampieri R et. al	0.6729	0.4572	2.1%	1.96 [0.80, 4.80]
He C et. al	-0.3696	0.1641	5.7%	0.69 [0.50, 0.95]
Hoshimoto S et .al	0.5253	0.408	2.5%	1.69 [0.76, 3.76]
Huh G et. al	0.885	0.2393	4.4%	2.42 [1.52, 3.87]
Yugawa K et. al	-0.1393	0.1037	6.8%	0.87 [0.71, 1.07]
Zhang C et.al	-0.4005	0.167	5.7%	0.67 [0.48, 0.93]
Zhao J et .al	-0.004	0.0036	7.8%	1.00 [0.99, 1.00]
**Subtotal (95% CI)**			**35.0%**	**1.02 [0.81, 1.30]**
Heterogeneity: Tau² = 0.06; Chi² = 29.97, df = 6 (P < 0.0001); I² = 80%				
Test for overall effect: Z = 0.19 (P = 0.85)				
**Total (95% CI)**			**100.0%**	**0.82 [0.71, 0.96]**
Heterogeneity: Tau² = 0.08; Chi² = 140.42, df = 20 (P < 0.00001); I² = 86%				
Test for overall effect: Z = 2.55 (P = 0.01)				
Test for subgroup differences: Chi² = 3.96, df = 1 (P = 0.05), I² = 74.7%				

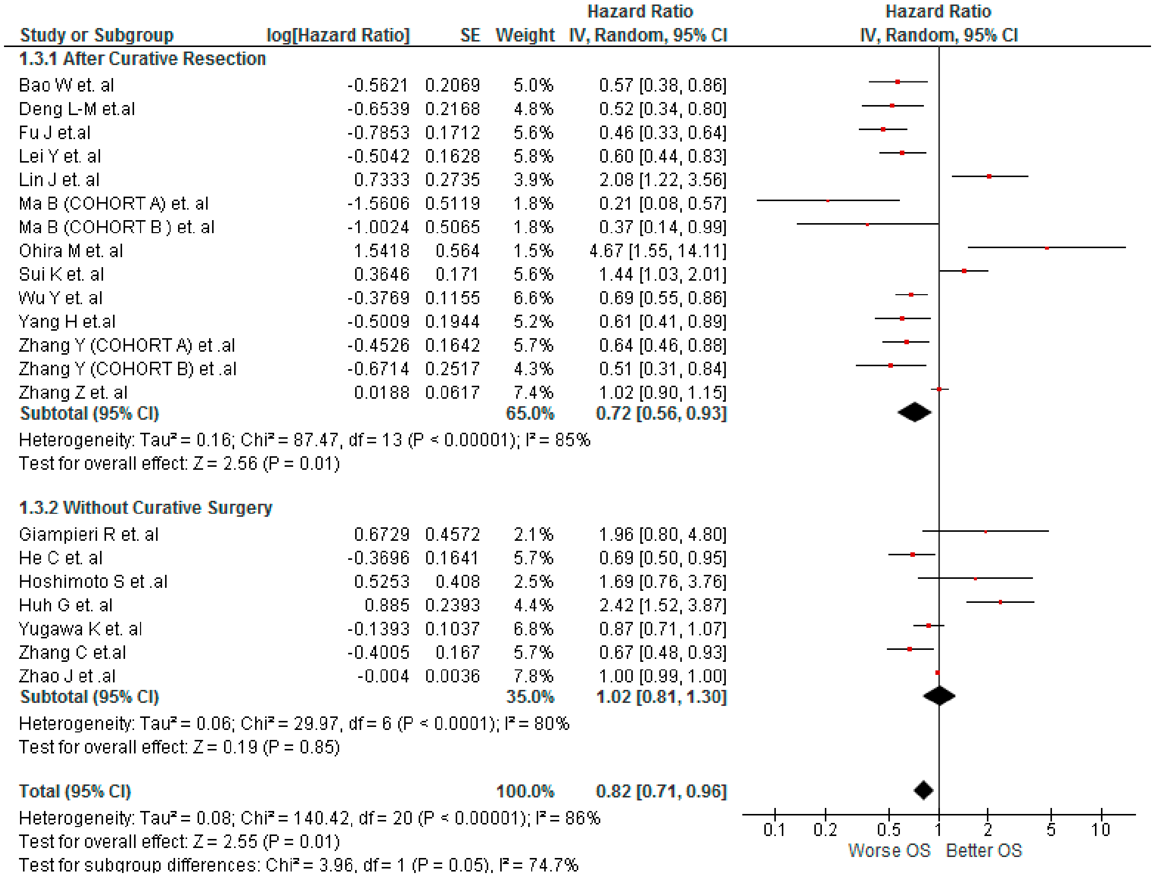

Figure S3. Subgroup analysis according to the origin country of the association between LMR and OS in patients with cholangiocarcinoma

Study or Subgroup	log[Hazard Ratio]	SE	Weight	Hazard Ratio IV, Random, 95% CI
**1.4.1 China**				
Bao W et. al	-0.5621	0.2069	5.0%	0.57 [0.38, 0.86]
Deng L-M et.al	-0.6539	0.2168	4.8%	0.52 [0.34, 0.80]
Fu J et.al	-0.7853	0.1712	5.6%	0.46 [0.33, 0.64]
He C et. al	-0.3696	0.1641	5.7%	0.69 [0.50, 0.95]
Lei Y et. al	-0.5042	0.1628	5.8%	0.60 [0.44, 0.83]
Lin J et. al	0.7333	0.2735	3.9%	2.08 [1.22, 3.56]
Ma B (COHORT A) et. al	-1.5606	0.5119	1.8%	0.21 [0.08, 0.57]
Ma B (COHORT B) et. al	-1.0024	0.5065	1.8%	0.37 [0.14, 0.99]
Wu Y et. al	-0.3769	0.1155	6.6%	0.69 [0.55, 0.86]
Yang H et.al	-0.5009	0.1944	5.2%	0.61 [0.41, 0.89]
Zhang C et.al	-0.4005	0.167	5.7%	0.67 [0.48, 0.93]
Zhang Y (COHORT A) et .al	-0.4526	0.1642	5.7%	0.64 [0.46, 0.88]
Zhang Y (COHORT B) et .al	-0.6714	0.2517	4.3%	0.51 [0.31, 0.84]
Zhang Z et. al	0.0188	0.0617	7.4%	1.02 [0.90, 1.15]
Zhao J et .al	-0.004	0.0036	7.8%	1.00 [0.99, 1.00]
**Subtotal (95% CI)**			**77.1%**	**0.68 [0.57, 0.81]**
Heterogeneity: Tau² = 0.08; Chi² = 108.78, df = 14 (P < 0.00001); I² = 87%				
Test for overall effect: Z = 4.41 (P < 0.0001)				
**1.4.2 Japan**				
Hoshimoto S et .al	0.5253	0.408	2.5%	1.69 [0.76, 3.76]
Ohira M et. al	1.5418	0.564	1.5%	4.67 [1.55, 14.11]
Sui K et. al	0.3646	0.171	5.6%	1.44 [1.03, 2.01]
Yugawa K et. al	-0.1393	0.1037	6.8%	0.87 [0.71, 1.07]
**Subtotal (95% CI)**			**16.4%**	**1.46 [0.87, 2.46]**
Heterogeneity: Tau² = 0.19; Chi² = 15.07, df = 3 (P = 0.002); I² = 80%				
Test for overall effect: Z = 1.45 (P = 0.15)				
**1.4.3 Italy**				
Giampieri R et. al	0.6729	0.4572	2.1%	1.96 [0.80, 4.80]
**Subtotal (95% CI)**			**2.1%**	**1.96 [0.80, 4.80]**
Heterogeneity: Not applicable				
Test for overall effect: Z = 1.47 (P = 0.14)				
**1.4.4 South Korea**				
Huh G et. al	0.885	0.2393	4.4%	2.42 [1.52, 3.87]
**Subtotal (95% CI)**			**4.4%**	**2.42 [1.52, 3.87]**
Heterogeneity: Not applicable				
Test for overall effect: Z = 3.70 (P = 0.0002)				
**Total (95% CI)**			**100.0%**	**0.82 [0.71, 0.96]**
Heterogeneity: Tau² = 0.08; Chi² = 140.42, df = 20 (P < 0.00001); I² = 86%				
Test for overall effect: Z = 2.55 (P = 0.01)				
Test for subgroup differences: Chi² = 33.27, df = 3 (P < 0.00001), I² = 91.0%				

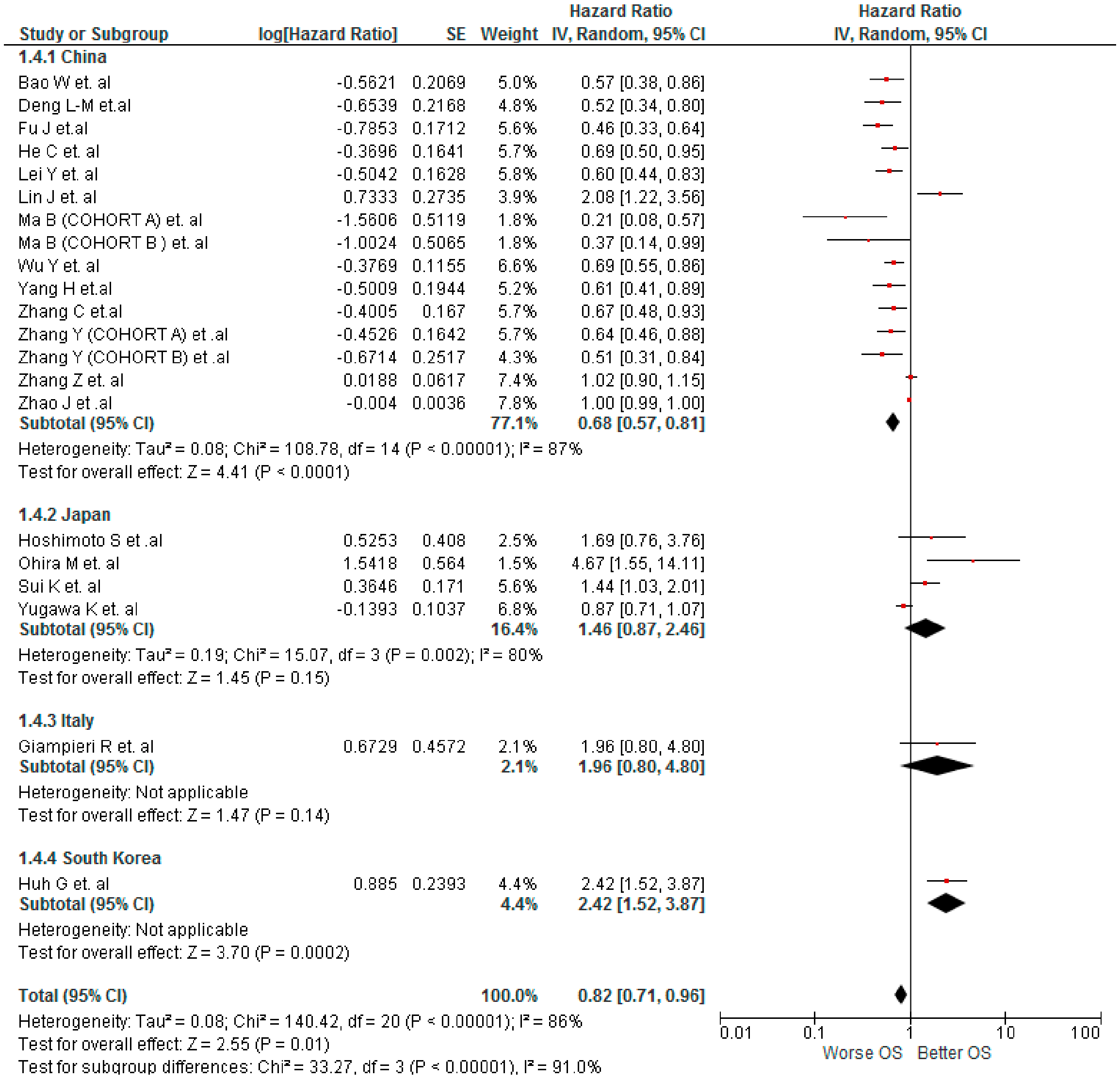

Figure S4. Sensitivity analysis according to risk of bias of the association between LMR and OS in patients with cholangiocarcinoma

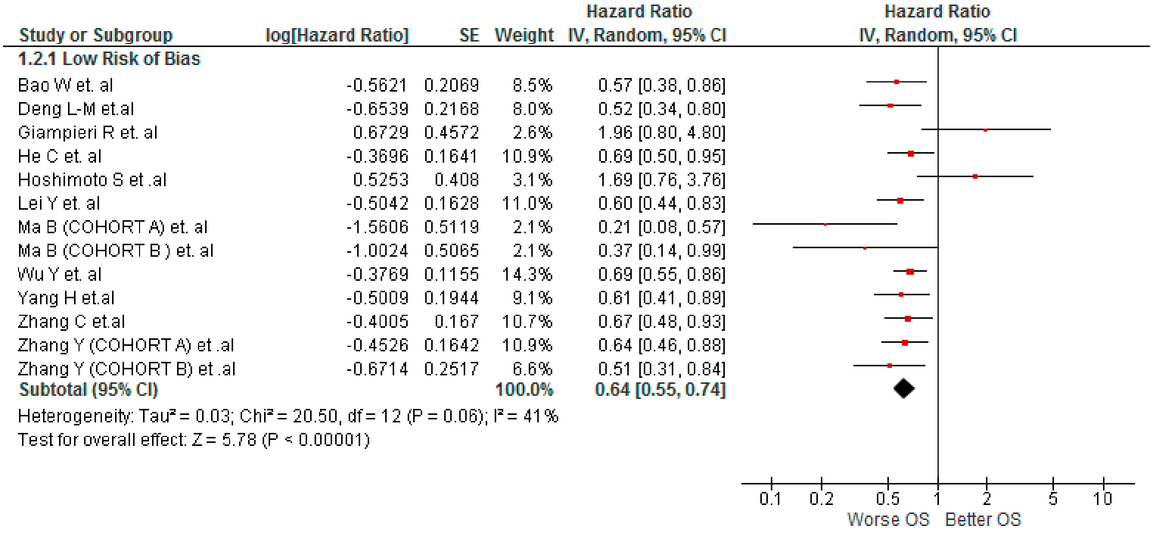

Figure S5. Funnel Plot of the studies that evaluated the association between LMR values and OS

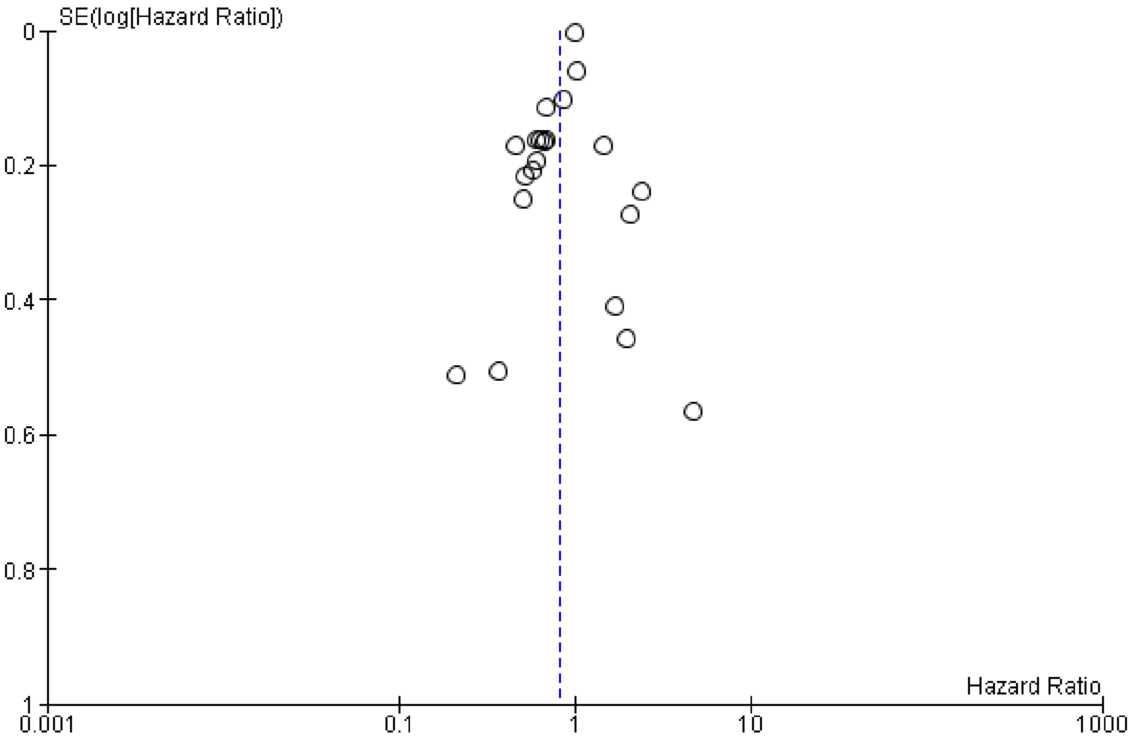

Supplement: Supplementary file 1 [file diagnostics-12-02655-s001.zip › diagnostics-1932344-supplementary.pdf]
